# Accession-specific modifiers act with ZWILLE/ARGONAUTE10 to maintain shoot meristem stem cells during embryogenesis in Arabidopsis

**DOI:** 10.1186/1471-2164-14-809

**Published:** 2013-11-20

**Authors:** Matthew R Tucker, Farshad Roodbarkelari, Elisabeth Truernit, Nikolai M Adamski, Annika Hinze, Barbara Lohmüller, Tobias Würschum, Thomas Laux

**Affiliations:** BIOSS Centre for Biological Signalling Studies, Faculty of Biology, Albert-Ludwigs-University Freiburg, Schänzlestrasse 1, 79104 Freiburg, Germany; Australian Research Council Centre of Excellence in Plant Cell Walls, University of Adelaide, Waite Campus, Urrbrae, SA 5064 Australia; State Plant Breeding Institute, University of Hohenheim, 70593 Stuttgart, Germany; ETH Zürich, LFW B32, Universitätsstr. 2, 8092 Zürich, Switzerland; John Innes Centre, Norwich Research Park, Norwich, NR4 7UH UK

**Keywords:** ZWILLE, ARGONAUTE, Shoot meristem, Stem cells, QTL, *Arabidopsis*, Accession

## Abstract

**Background:**

Stem cells located in the centre of the shoot apical meristem are required for the repetitive formation of new organs such as leaves, branches and flowers. In *Arabidopsis thaliana*, the *ZWILLE/PINHEAD/AGO10* (*ZLL*) gene encodes a member of the ARGONAUTE (AGO) protein family and is required to maintain shoot meristem stem cells during embryogenesis. In the Landsberg *erecta* (Ler) acession, *ZLL* is essential for stem cell maintenance, whereas in the Columbia (Col) accession its requirement appears masked by genetic modifiers. The genetic basis for this variation has remained elusive.

**Results:**

To understand the impact of natural variation on shoot stem cell maintenance, we analysed 28 wild-type *Arabidopsis* accessions from around the world and show that *ZLL* function is essential for stem cell maintenance in accessions mainly originating from Germany, but is dispensable for accessions from other regions. Quantitative Trait Loci (QTL) mapping using Ler/Col recombinant inbred lines indicated that at least five genomic regions, referred to as *FLETSCHE (FHE) 1*–*5*, modify *ZLL* function in stem cell maintenance. Characterisation of Col *zll* near isogenic lines confirmed that the major QTL, *FHE2*, is preferentially maintained as a Ler allele in seedlings lacking stem cells, suggesting that this region harbours an important modifier of *ZLL* function. Comparison of torpedo-stage embryo expression profiles to QTL map data revealed candidate *FHE* genes, including the *Arabidopsis* Cyclophilin-40 homologue *SQUINT* (*SQN*), and functional studies revealed a previously uncharacterised role for *SQN* in stem cell regulation.

**Conclusions:**

Multiple genetic modifiers from different *Arabidopsis* accessions influence the role of *ZLL* in embryonic stem cell maintenance. Of the five *FHE* loci modifying stem cell maintenance in Ler-0 and Col-0, *FHE2* was the most prominent and was tightly linked to the *SQN* gene, which encodes a cofactor that supports AGO1 activity. *SQN* shows variable embryonic expression levels between accessions and altered *ZLL*-dependency in transgenic assays, confirming a key role in stem cell maintenance. Reduced *SQN* expression levels in Col-0 correlate with transposon insertions adjoining the transcriptional start site, which may contribute to stem cell maintenance in other *ZLL*-independent accessions.

**Electronic supplementary material:**

The online version of this article (doi:10.1186/1471-2164-14-809) contains supplementary material, which is available to authorized users.

## Background

The shoot apical meristem (SAM) is a dynamic system that sustains production of plant organs while constantly integrating developmental and environmental cues [1, 2]. The ability of the SAM to process and buffer these cues is already evident during early stages of embryogenesis, when signals from different embryonic domains, including the vasculature and the epidermis, are integrated to qualitatively influence meristem growth [3, 4]. Many of these signals feed into a core negative feedback loop involving the transcription factor *WUSCHEL* (*WUS*) and the *CLAVATA1/2/3* (*CLV*) signaling complex, which act within the meristem to balance stem cell maintenance and cell differentiation [5–7].

In *Arabidopsis*, mutations in *AGO1* and *ZLL* influence stem cell maintenance in the embryonic meristem and also during subsequent growth [8–11]. The relationship between these two genes is complex, with studies indicating both synergistic and antagonistic functions [11–14]. In general, AGO proteins act as key mediators of small RNA (sRNA) silencing pathways by binding 21–24 nt sRNAs and inducing silencing of complementary RNA or DNA targets [15]. Recent biochemical and genetic evidence suggests that in the embryo, ZLL acts as a miRNA “locker” to sequester microRNA165/6, thereby limiting its incorporation into the active AGO1 RNA-Induced Silencing Complex (RISC) [12, 16]. In the absence of *ZLL* function, AGO1 is proposed to bind miR165/6 and down-regulate Class III HD-ZIP transcription factors within the embryonic SAM, thereby inducing stem cell differentiation [17]. This pathway likely influences function of WUS in promoting stem cell identity, since WUS-induced *CLV3* expression in stem cells is disrupted in *zll* mutants [10]. *ZLL* function also appears to be linked to vascular tissues because provascular *ZLL* expression in the embryo is sufficient to maintain stem cell development, indicating that movement of small RNAs or other signaling molecules may be involved [10].

**Figure 1:**
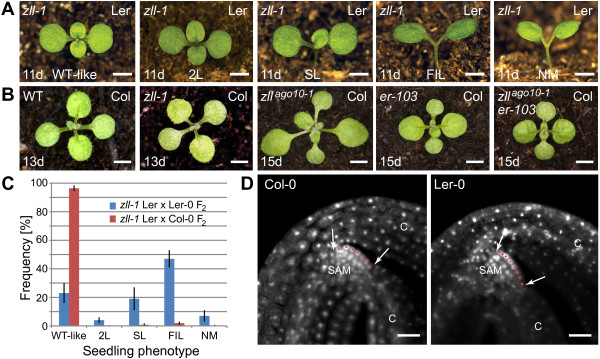
**Shoot meristem development in** ***Arabidopsis*** **wild-type and** ***zll*** **mutants. A.** Stem cell termination phenotypes in *zll-1* Landsberg *erecta* (Ler) mutants. Seedlings show either a wild-type-like meristem (WT-like), stem cell termination after the formation of two leaves (2L), a single central leaf-like organ (SL), a single central filament (FIL) or a flat apex without any organ formation (NM). Bar = 2.5 mm. **B.** Most *zll-1* Columbia-0 (Col) seedlings cannot be phenotypically discerned from Col WT. Similarly, the *zll* ^*ago10-1*^ Col mutant appears WT-like in the vast majority of seedlings and stem cell termination is not enhanced by mutations in the Col *ERECTA* (*ER*) gene, such as *er-103*. Bar = 2.5 mm. **C.** Frequency of stem cell termination phenotypes in the F_2_ progeny of crosses between Ler *zll-1*, Ler-0 and Col-0. Abbreviations as per **A. D.** Mature embryos stained with propidium iodide show subtle differences in the number of L1 cells (red dots) between Ler-0 and Col-0. The width of the meristem is SAM, shoot apical meristem, C, cotyledon indicated with arrows. Bar = 25 μm.

One intriguing aspect of the ZLL regulatory pathway is that *zll* mutants show SAM stem cell defects in an accession-specific manner. While *zll* alleles isolated in the Ler background show premature termination of stem cells [8, 13], putative null *zll* alleles in the Col background, such as *zll*^*ago10-1*^[11, 18], have no or minimal effects (Figure 1). Furthermore, putative homozygous null mutants in Ler display a variable expressivity of stem cell termination [10]. Phenotypes range from an empty apex to a filamentous structure, a single leaf or two leaves in place of the SAM, together with a fraction of individuals that develop a fully functional shoot meristem (Figure 1A) [8]. All of these mutant seedlings eventually produce adventitious meristems, flowers and seed, allowing them to be propagated and crossed as homozygotes. In an EMS screen for modifiers of the Col *zll*^*ago10-1*^ allele, several genes and pathways that enhance *ZLL* function were identified, including miR394 [4]. This suggests that activity of multiple pathways can compensate for loss of ZLL activity. Natural variation in early meristem development has previously been noted in maize, where *knotted1* loss-of-function alleles show different degrees of embryonic meristem development in different inbred backgrounds [19].

In this study we examined the effect of different genomic regions from Ler and Col on ZLL function in shoot stem cell maintenance. QTL analysis indicates that at least five loci, referred to as *FLETSCHE* (*FHE*) *1*–*5*, influence stem cell maintenance. Comparison of embryo transcriptomic profiles identified multiple genes showing variable expression in different accessions, including candidates for the *FHE* loci. One of the candidates for *FHE2* represents an allele of *SQN*, which encodes the *Arabidopsis* Cyclophilin-40 orthologue and acts as a modifier of ZLL function.

## Results

### *ZLL* is required for meristem maintenance in an accession specific manner

The frequency of homozygous seedlings showing shoot stem cell termination in Ler *zll* alleles varies from 10 to 90%, depending on the mutation [8, 13, 20]. By contrast, the putative null T-DNA insertion mutants *zll*^*ago10-1*^ and *zll*^*ago10-3*^ in the Col accession have no or minimal effects on stem cell maintenance and meristem development (~0.2% in *zll*^*ago10-1*^[11, 18]). To test if this difference is related to the nature of the respective mutant alleles, *zll*^*ago10-1*^ was backcrossed three times to Ler-0 wild-type. In homozygous Ler *zll*^*ago10-1*^ lines, 29% (*n* = 194) of the seedlings showed stem cell termination. In a converse experiment, the strong *zll-1* EMS mutant allele was introduced into the Col background by crossing Ler *zll-1* to Col-0 wild-type. Only a small fraction (0.5%, *n* = 5736) of the expected 25% *zll-1* homozygous F_2_ seedlings showed defects in stem cell maintenance (Figure 1B, C). This equates to an approximate phenotype of 2% in the homozygous state, compared to 61% (*n* = 315) in a cross between Ler *zll-1* and Ler-0. This indicates that the different expressivity of the *zll-1* mutation between Ler-0 and Col alleles is not due to the nature of the mutant alleles, but must be caused by genetic modifiers. To determine if the different requirement for *ZLL* between Ler and Col might be due to the *erecta* mutation in Ler, the Col *zll*^*ago10-1*^ allele was crossed to the strong *er-102* and intermediate *er-103* Col alleles (Figure 1B) [21]. Double mutants showed the characteristic *erecta* phenotype, but did not show an increased frequency of stem cell defects compared to the *zll*^*ago10-1*^ single mutant.

Differences in embryonic meristem size between Ler and Col were also investigated as a possible explanation for the different expressivity of *zll* mutations. Because the number of L1 cells in the mature embryonic meristem is indicative of the size of the meristem, wild-type embryos from the Ler-0 and Col-0 accessions were stained with propidium iodide and examined by confocal laser microscopy (Figure 1D). Grown under the same conditions, Ler-0 embryos (*n* = 17) contained ~11.4 (SD +/-1.4) cells in the L1 layer of the meristem at maturity, while Col-0 embryos (*n* = 25) contained ~10.1 (SD +/-1.5) cells. This indicates that the L1 layer of the Ler-0 embryonic meristem, and hence the meristem itself, contains more cells than the Col-0 meristem (Students *t*-test P < 0.01). This opposes the hypothesis that a smaller meristem in Ler-0 might be the cause of increased sensitivity to *zll* mutations, but suggests that fundamental differences in embryonic meristem regulation may contribute to variable *zll* expressivity in these accessions.

### *ZLL* is required for meristem maintenance in multiple Arabidopsis accessions

**Figure 2:**
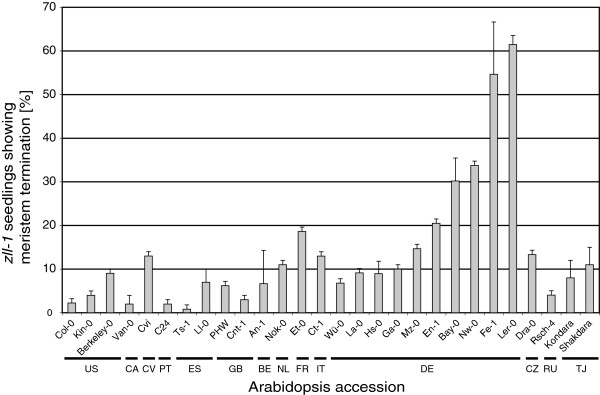
**Shoot meristem development in F**_**2**_ **progeny from 28 accessions of** ***Arabidopsis*** **crossed to Ler** ***zll-1.*** Columns present the proportion of *zll-1* homozygous seedlings showing a stem cell termination phenotype. Error bars show standard deviation after four independent seedling counts. The country of origin for each accession is indicated. US, United States of America, CA, Canada, CV, Cape Verde Islands, PT, Portugal, ES, Spain, GB, United Kingdom, BE, Belgium, NL, Netherlands, FR, France, IT, Italy, DE, Germany, CZ, Czech Republic, RU, Russia, TJ, Tajikistan.

**Figure 3:**
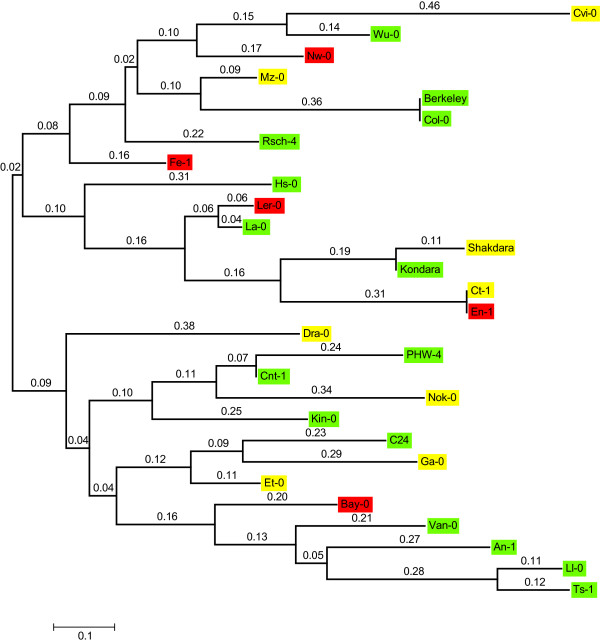
**Molecular phylogenetic relationships between 28** ***Arabidopsis*** **accessions based on 59 single nucleotide polymorphisms.** Genotypes were extracted from http://www.naturalvariation.org/. The evolutionary history was inferred by using the Maximum Likelihood method based on the Tamura-Nei model. The tree with the highest log likelihood (-1257.96) is shown and was generated in MEGA5.2. The tree is drawn to scale, with branch lengths measured in the number of substitutions per site (above the branches). All positions containing gaps and missing data were eliminated. Red shading indicates a capacity for a high frequency of *zll*-dependent stem cell termination, yellow indicates a medium capacity and green indicates a low capacity.

To determine if the differences in *ZLL*-dependency for stem cell maintenance are restricted to Ler-0 and Col-0, the Ler *zll-1* allele was crossed to 28 different wild-type *Arabidopsis* accessions originating from diverse countries (Figure 2). These accessions and others were analysed previously with 149 single nucleotide polymorphisms (SNPs) to address *Arabidopsis* population structure [22]. Analysis of 59 SNPs that produced clear genotypes in the 28 accessions used here confirmed that apart from the pairs of Berkley and Col-0, and Ct-1 and En-1, the accessions were different (Figure 3).

The frequency of seedling meristem termination, indicative of stem cell defects during embryogenesis, was assessed in the F_2_ generation of each accession cross to determine the approximate phenotypic frequency in the homozygous *zll-1* state (Figure 2). Of the 28 accessions, five showed stem cell termination in more than 20% of the homozygous seedlings, eight showed a phenotype in 10 to 20%, and fifteen showed a phenotype in less than 10% of seedlings. Curiously, the five accessions that showed the highest *zll-1* expressivity, Ler-0, Freiburg-1 (Fe-1), Neuweilnau-0 (Nw-0), Bayreuth-0 (Bay-0) and Enkheim-1 (En-1), were all derived from locations in the southern half of Germany. Despite this geographical association, there is no obvious clustering of these accessions in a phylogenetic tree based on 59 SNPs to suggest they were more related to each other than accessions showing weak stem cell termination phenotypes (Figure 3).

### Multiple quantitative trait loci (QTL) influence *ZLL*-dependent stem cell maintenance

**Figure 4:**
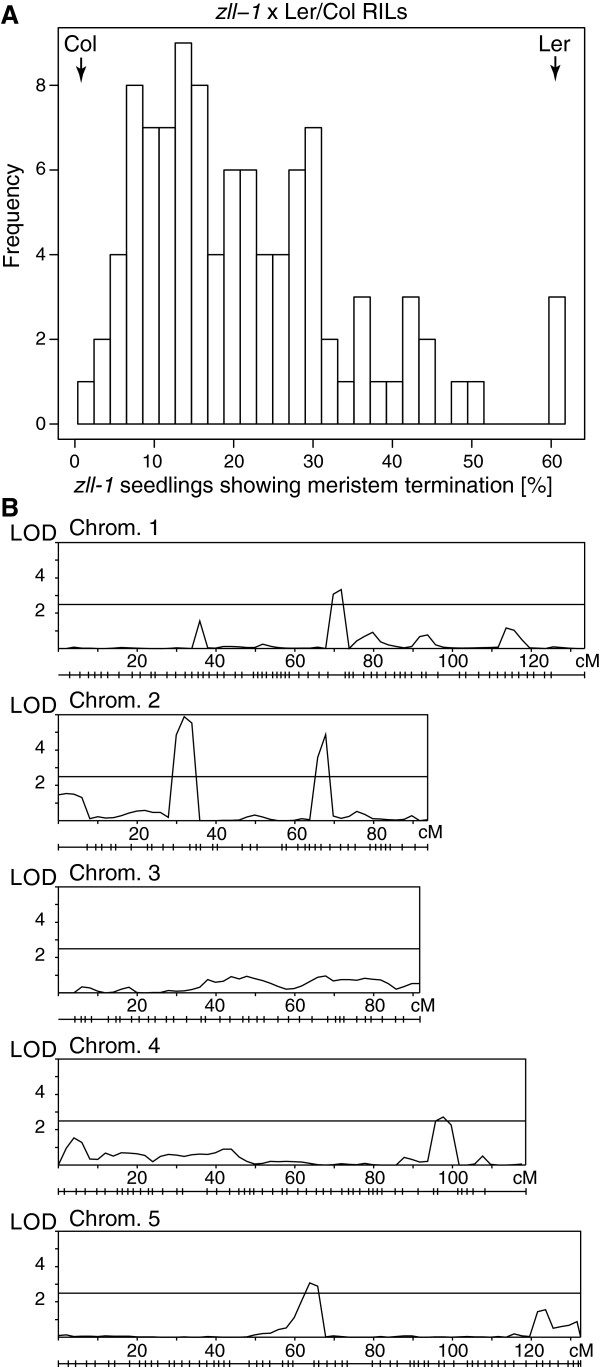
**QTL analysis of** ***zll-1*** **x Ler/Col Recombinant Inbred Lines. A.** Frequency distribution plot of stem cell termination phenotypes in the F_2_ progeny of 101 crosses between Ler *zll*-*1* and 99 RILs, Ler-0 and Col-0. **B.** Chromosome-wide Logarithm of the Odds (LOD) scores of QTL influencing stem cell termination. The horizontal line indicates the significance threshold and marker positions are shown below each plot. LOD values and significance thresholds were determined using PlabMQTL software.

To locate candidate modifiers in the *Arabidopsis* genome, we utilized a population of Ler/Col Recombinant Inbred Lines (RILs) [23]. Each line contains a different combination of Ler-0 and Col-0 genomic regions and has been genotyped, providing an excellent resource for mapping QTL. The Ler *zll-1* allele was crossed to 99 RILs and the two parents, and F_2_ progeny were scored for stem cell termination phenotypes (Figure 4A). The expectation from this cross was that RILs containing Ler-0 alleles at the position of putative QTL would show a higher frequency of seedlings with stem cell defects than lines containing Col-0 alleles at the same position.

On average, 684 F_2_ seedlings were scored for stem cell defects in each RIL x Ler *zll-1* cross and the frequency was multiplied by 4 to determine the approximate frequency within the homozygous *zll-1* population. Values ranged from 1% to 61% (Figure 4A; Additional file 1) and the frequency of RIL seedlings showing stem cell defects was used as the phenotype for QTL mapping. This identified five QTL (Figure 4B, Table 1), which are hereafter referred to as the *FLETSCHE (FHE) 1–5* loci (German synonym for *ZWILLE*). The proportion of variance explained by the individual *FHE* loci ranged from 7.7 to 15.9% and in total, the five *FHE* QTL explained 49% of the variance. The individual QTL effects ranged from 3.63 to 6.12 percent shoot termination and Ler-0 always contributed the allele increasing the frequency of seedlings showing stem cell defects. The largest effect QTL was *FHE2*, located on Chromosome 2 at 32 cM.

**Table 1:** **Detection of** ***FLETSCHE*** **(** ***FHE*** **) QTL in the Ler/Col RIL population**

QTL	Chr	Pos	LOD	***p***	α-effect
*FHE1*	1	72	3.45	8.6	3.85
*FHE2*	2	32	6.83	15.9	6.12
*FHE3*	2	68	3.47	8.7	4.10
*FHE4*	4	98	3.24	8.1	3.70
*FHE5*	5	64	3.03	7.7	3.63
Total				49.0	

Chr = Chromosome number, Pos = QTL position in centiMorgan, LOD = Logarithm of the odds score, *p* = proportion of explained variance in % and α = the allele substitution effect for the allele originating from Ler.

### Near Isogenic *zll-1* Lines (NILs) showing shoot stem cell defects preferentially retain genomic regions linked to the Ler *FHE* loci

**Figure 5:**
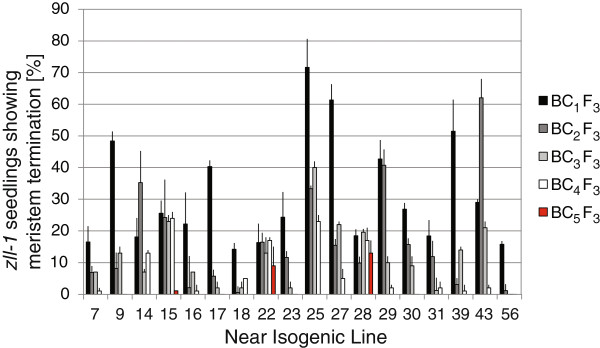
**Shoot meristem development in F3 progeny from 18** ***zll-1*** **Col introgression lines.** The frequency of seedlings showing stem cell termination phenotypes in each *zll-1* near isogenic line (NIL) is shown. Error bars show standard deviation after three replicate seedling counts.

To further assess the contribution of *FHE*^Ler^ loci to stem cell maintenance, the *zll-1* mutation was introgressed from Ler to Col. Twenty independent F_2_ plants showing stem cell defects in the first backcross of Ler *zll-1* to Col-0 (termed BC_1_F_2_) were further backcrossed to Col-0 wild-type three to six times. In each backcross, F_2_ plants that showed stem cell defects at the seedling stage were selected for further backcrossing. At the same time, F_3_ seeds from self-fertilised siliques were harvested to assess the frequency of stem cell defects in the progeny. Each backcross was expected to remove non-essential Ler DNA, while retaining Ler genomic regions that enhance *zll* stem cell termination phenotypes. Although some lines showed relatively stable levels of stem cell defects over several backcrosses (Figure 5), the majority showed a decrease with each subsequent backcross (the average phenotypic frequency of all lines decreased from 31 ± 16% after BC_1_ to 13 ± 8% after BC_3_), consistent with the gradual accumulation of Col-0 modifiers suppressing stem cell termination. This gradual decrease strongly indicates that multiple loci suppress stem cell termination in the Col-0 accession in a quantitative manner.

Two lines (NIL22 and NIL28) that maintained relatively high frequencies of stem cell termination over several backcrosses were selected for mapping and phenotypic analysis after BC_5_. Genomic DNA from phenotypic NIL22 BC_5_F_2_ plants (*n* = 48) and NIL28 BC_5_F_2_ plants (*n* = 48) was used for bulk mapping with 19 PCR-based markers (Additional file 2) that detect Ler/Col polymorphisms in the vicinity of the predicted *FHE* QTL and at unlinked sites throughout the genome. In both NILs, PCR results identified a strong bias towards Ler DNA at markers MT435 (~32 cM) and nga168 (~73 cM) on chromosome 2 and ciw9 (~90 cM; *zll-1*) on chromosome 5, while the rest of the genome was biased towards Col.

Subsequent mapping of all individual phenotypic NIL22 and NIL28 BC_5_F_2_ plants revealed that a large region of genomic DNA encompassing both *FHE2* and *FHE3*, approximately 40 cM in length, was preferentially maintained as homozygous or heterozygous Ler (153/192 chromosomes at marker MT435, *p* = 1.92e-16; 148/192 chromosomes at nga361, *p* = 6.12e-14) indicative of segregation distortion. A limited number of BC_5_F_2_ plants containing smaller regions of Ler DNA around the *FHE2* and *FHE3* loci were identified, and these were analysed in the BC_5_F_3_ to determine the specific effect of the Ler *FHE2* locus on stem cell termination (Table 2). Although homozygous Ler DNA at both *FHE2* and *FHE3* was not absolutely essential for stem cell termination, such plants showed a higher frequency of stem cell termination (10 ± 4% n = 554) in the BC_5_F_3_ compared to any other combination of *FHE2/FHE3* DNA (Table 2). Plants containing Ler DNA at *FHE2* but not *FHE3* (i.e. *FHE2*^Ler/Ler^*FHE3*^Col/Col^*zll-1*), showed stem cell termination in 5 ± 2% (*n* = 388) of homozygous *zll-1*seedlings. This is similar to the allele substitution effect estimated for *FHE2*^Ler^ in the Ler/Col RIL population of 6.1%. By contrast, no plants showing stem cell termination were identified containing homozygous Ler DNA only at *FHE3* (i.e. *FHE2*^Col/Col^*FHE3*^Ler/Ler^*zll-1*). Of the phenotypic progeny derived from *FHE2*^Ler/Col^*zll-1* parents, 70% (*n* = 156) became homozygous *FHE2*^Ler/Ler^*zll-1* in the BC_5_F_3_ generation compared with the expected 25% for random segregation. Phenotypic *FHE2*^Ler/Col^*FHE3*^Ler/Col^*zll-1* plants could only be identified at low frequency (1 ± 1% *n* = 536), and no phenotypic plants containing homozygous Col DNA at both *FHE2* and *FHE3* could be detected. Collectively, these data suggest that the *FHE2*^Ler^ and to a lesser extent the *FHE3*^Ler^ loci encode quantitative modifiers of the *zll* stem cell termination phenotype.

**Table 2:** **Effect of** ***FHE2*** **and** ***FHE3*** **genomic regions on stem cell termination in BC**_**5**_ ***zll-1*** **Col-0 introgression lines**

Genotype category	Introgression line (F_2_plant number)	***FHE2***genotype	***FHE3***genotype	Frequency of stem cell termination in F_3_progeny ± SD
1	NIL28.5 (#8, #16)	Ler	Ler	10 ± 4% (*n* = 554)
2	-	Ler	Het	n.d.
3	NIL28.5 (#9) NIL22.5 (#3)	Het	Ler	9 ± 1% (*n* = 783)
4	-	Col	Ler	n.d.
5	NIL22.5 (#7)	Ler	Col	5 ± 2% (*n* = 288)
6	NIL28.5 (#14, #15)	Het	Het	1 ± 2% (*n* = 536)
7	-	Het	Col	n.d.
8	NIL28.5 (#13, #27)	Col	Het	1 ± 0% (*n* = 902)
9	-	Col	Col	n.d.

NIL = Near Isogenic Line, SD = standard deviation, Het = Heterozygous, *n* = total seedlings analysed, - = no plants identified with the corresponding genotype, n.d. = not determined.

### Changes in gene expression between *ZLL*-independent and dependent accessions identify candidates for the *FHE* loci and downstream pathways

Despite further backcrosses, most *zll-1* Col NILs preferentially retained a large fragment of Ler genomic DNA around *FHE2*^Ler^ which complicated fine mapping. To further delineate putative *FHE* factors and pathways that influence stem cell maintenance in a *ZLL*-dependent manner, microarray profiles were generated from specific *ZLL*-dependent and independent *Arabidopsis* accessions using Affymetrix ATH1 chips.

Torpedo-stage embryos were harvested separately from four *ZLL*-dependent accessions (Ler-0, Fe-1, Nw-0, and Bay-0), and three *ZLL*-independent accessions (Col-0, Van-0 and Ts-1). Consistent with previous SNP genotyping results [22], none of the accessions showed an identical pattern when genotyped with 14 INDEL markers, confirming that they are genetically unique. Multiple comparisons were made between expression profiles derived from the two groups of accessions by maintaining a minimum of three arrays per group. A total of 439 genes were identified as being differentially expressed in at least one of the comparisons, based on a p-value of <0.05 and a 3-fold expression change (Additional file 3). In the most stringent comparison, all of the *ZLL*-dependent accessions were grouped as replicates and compared to the *ZLL*-independent accessions. Two genes were identified as being up-regulated and ten were identified as being down-regulated (Additional file 4). None of the genes appeared to be tightly linked to the predicted *FHE* loci from the RIL analysis, suggesting that they may either represent factors that act downstream of the *FHE* modifiers, they are unrelated to ZLL function, and/or they represent modifiers that escaped detection in the Ler/Col *FHE* QTL mapping.

### *FHE2* maps close to the Arabidopsis Cyclophilin-40 homologue *SQUINT*, which is differentially expressed between Col-0, Fe-1 and Ler-0 accessions

Expression profiles from Col-0, Fe-1 and Ler-0 were examined in greater detail to delineate putative *FHE* loci, because: (1) the QTL mapping data were derived from Ler-0 and Col-0, and (2) Fe-1 does not contain the *erecta* mutation, but it is the most likely accession to share similar *FHE* modifiers with Ler-0 based on its high frequency of stem cell termination after *zll-1* introgression. A total of 184 genes were differentially expressed between Col-0 and Fe-1/Ler-0 (Additional file 3). These were sorted based on their chromosomal position and compared to lists of genes residing approximately 5 cM either side of the putative *FHE* loci (Table 3). On chromosome 2, nineteen genes were identified that showed differential expression. Four of these were located in the vicinity of the *FHE2* QTL. Three of the genes (At2g13790, At2g14800 and At2g15325) were expressed at ~3 to 7 fold lower levels in Fe-1/Ler-0 compared to Col-0, while the fourth gene (At2g15790) showed ~5 fold higher expression in Fe-1/Ler-0 compared to Col-0. The three down-regulated genes encode a transposable element protein, an unknown protein and a pathogenesis-related lipid transfer protein, respectively. The fourth gene, which was located at the predicted physical position of the *FHE2* QTL, encodes the *Arabidopsis* homologue of Cyclophilin-40, also known as SQUINT (SQN [24]). *SQN* is involved in several stages of plant development, including the transition from juvenile to adult phase in the shoot meristem [24] and floral meristem termination [25], and has also been identified as a factor required for function of AGO1 [26]. In the absence of *SQN*, mRNA levels of miRNA-regulated genes are increased and weak *ago1* hypomorphic alleles resemble *ago1* nulls, suggesting that the two proteins cooperate in the same pathway [26].

**Table 3:** ***Arabidopsis*** **genes differentially expressed in torpedo stage embryos from Col-0 and Fe-1/Ler-0 and located in the vicinity of predicted** ***FHE*** **QTL**

Predicted QTL	Gene	Affy ID	Description	Col vs Fe/Ler (FC)	Predicted QTL
***FHE1***	AT1G43780	260859_at	Serine carboxypeptidase-like 44	-11.1	0.008
	AT1G48180	257493_at	unknown protein	-3.7	0.038
	AT1G50520	261879_at	CYP705A27 (cytochrome P450)	4.1	0.001
***FHE2***	AT2G13970	265302_at	transposable element gene	7.2	0.000
	AT2G14800	267110_at	unknown protein	3.8	0.010
	AT2G15325	257438_at	Lipid transfer protein (LTP)	5.6	0.007
	AT2G15790	265483_at	SQUINT Cyclophilin-40	-5.2	0.000
***FHE3***	AT2G33220	245169_at	similar to MEE4	5.8	0.000
	AT2G33790	267457_at	pollen Ole-e1 allergen	-5.5	0.001
	AT2G35820	263947_at	unknown protein	-3.1	0.002
	AT2G36550	263910_at	similar to NLI interacting factor	-9.3	0.001
***FHE4***	AT4G39190	252938_at	GNS1/SUR4 membrane protein	-3.4	0.017
***FHE5***	AT5G36910	249645_at	THIONIN 2.2	16.5	0.002
	AT5G38580	249517_at	F-box family protein	-3.2	0.014
	AT5G38700	249522_at	unknown protein	3.2	0.011
	AT5G38960	249479_at	germin-like protein, putative	6.2	0.003
	AT5G39060	258246_s_at	transposable element gene	17.0	0.000
	AT5G39100	249495_at	GERMIN-LIKE PROTEIN 6	-3.8	0.042
	AT5G39210	249472_at	CRR7	4.8	0.044
	AT5G41650	249258_at	glyoxalase I family protein	4.2	0.002
	AT5G42280	249645_at	DC1 domain-containing protein	3.9	0.000

Affy ID = Affymetrix gene chip identifier, FC = fold change >3.0.

### Changes in *SQN* expression influence stem cell maintenance in the Ler-0 and Col-0 accessions

Based on the antagonistic roles of AGO1 and ZLL in stem cell regulation, we speculated that increased *SQN* expression in Ler-0 might enhance AGO1 activity. In the absence of *ZLL* this could lead to stronger repression of AGO1 targets (i.e. Class III HD-ZIP genes) and subsequent stem cell termination. To test this model, we examined whether reduced *SQN* expression could alleviate meristem defects in Ler *zll-1* by crossing to the Ler *sqn-4* allele [25]. F_2_ plants homozygous for both *sqn-4* and *zll-1* were identified by PCR genotyping and their progeny was examined at the seedling stage. Compared to *zll-1* siblings, *zll-1 sqn-4* double mutants displayed weaker stem cell termination phenotypes (Table 4). In *zll-1* plants, 56% of seedlings terminated with a single filamentous structure, while 18% terminated with one or two leaves. In contrast, only 34% of *zll-1 sqn-4* plants terminated with a single filament, while 58% terminated with one or two leaves.

**Table 4:** **Analysis of** ***amiRSQN*** **in Ler** ***zll-1 sqn-4*** **double mutants**

		Stem cell termination phenotypes in %				
**Line**	***n***	**NM**	**FIL**	**SL**	**2L**	**WT-like**
*zll-1 SQN* ^*+/+*^	522	16.1	55.7	15.7	2.7	9.8
*zll-1 sqn-4*	184	1.1	34.1	43.4	14.3	8.2
*zll-1 sqn-4 amiRSQN#1*	395	0.0	24.3	19.0	37.2	19.5
*zll-1 sqn-4 amiRSQN#2*	382	0.0	11.8	15.7	41.4	31.2
*zll-1 sqn-4 amiRSQN#3*	156	0.6	16.0	39.1	28.2	16.0
*zll-1 sqn-4 amiRSQN#4*	363	0.3	17.4	44.1	14.9	23.4
*zll-1 sqn-4 amiRSQN#5*	152	0.0	15.1	49.3	17.8	17.8
*zll-1 sqn-4 amiRSQN#6*	411	0.0	2.7	48.2	17.8	31.1
*zll-1 sqn-4 amiRSQN#7*	348	0.0	8.6	40.8	33.3	17.0

*n* = total seedlings counted, NM = no-meristem activity, FIL = filament, SL = single leaf-like structure, 2 L = two leaves, WT-like = wild-type like meristem

Because *sqn-4* is a weak allele that may only partially reduce SQN activity by modifying the C’-terminus of the predicted SQN protein [25], an artificial miRNA was designed to further down-regulate *SQN* mRNA levels. In *zll-1 sqn-4* double mutants, *amiRSQN* suppressed stem cell defects and enhanced the frequency of a wild type-like shoot meristem in seedlings up to 31.2% (Table 4). In a reciprocal experiment, to address whether lower levels of *SQN* in *ZLL*-independent accessions such as Col-0 may be a reason for the absence of stem cell defects, *SQN* was ectopically expressed from the strong embryonic *pAtRPS5a* promoter in the Col *zll*^*ago10-1*^ mutant. This construct induced stem cell termination in up to 22.2% of *zll*^*ago10-1*^ transgenic plants (Table 5). Taken together, these results suggest that different *SQN* expression levels in the Ler and Col accessions determine differences in stem cell termination in the absence of *ZLL* function, and that *SQN* is a strong candidate for a gene underlying the *FHE2* QTL.

**Table 5:** **Analysis of ectopic** ***SQN*** **expression in Col** ***zll*** ^***ago10-1***^ **mutants**

Line	***n***	Seedlings showing meristem termination [%]
*zll* ^*ago10-1*^	508	0.6
*zll* ^*ago10-1*^ *pAtRPS5a:SQN#1*	284	6.6
*zll* ^*ago10-1*^ *pAtRPS5a:SQN#3*	134	22.2

*n* = total seedlings counted.

## Discussion

### Different genetic backgrounds influence the role of *ZLL* in stem cell maintenance

Previous studies in *Arabidopsis* have utilised variation between natural accessions to identify the genetic basis for developmental differences [27, 28], including small RNA-mediated regulation of flowering time [29], self-incompatibility [30] and root growth [31]. In the current study, natural genetic modifiers that influence stem cell maintenance in the absence of ZLL function were investigated. The aim was to identify novel components that support ZLL in promoting stem cell maintenance through the regulation of WUS activity [10] and/or modification of miRNA function [12]. QTL mapping in a population of Ler/Col RILs and *zll-1* Col-0 NILs suggested that five *FHE* loci can explain 49% of the variance in stem cell maintenance in Ler-0 and Col-0. In all cases, the presence of the Ler *FHE* alleles with *zll-1* resulted in an increased frequency of stem cell termination, consistent with Col *zll* mutants showing limited degrees of stem cell termination. The Ler/Col RILs were previously analysed for variation in shoot regeneration from tissue culture, which depends on three QTL on chromosomes 1, 4 and 5 [32, 33]. The position of these loci is distinct from the *FHE* QTL, suggesting that the *FHE* loci are unlikely to be involved in shoot formation *per se,* and are more likely to be involved in embryonic meristem function.

Our findings also indicate that differences in *ZLL*-dependency are not limited to the Col-0 and Ler-0 accessions and considerable variation exists between different accessions from North America, Europe and Asia. The two accessions showing the highest frequency of stem cell defects in the presence of *zll-1*, in 55% and 61% of *zll-1* seedlings respectively, were Fe-1 and Ler-0. Limited information is available for the Fe-1 accession, but previous studies of natural genetic variation show that it diverges from Ler and Col in its response to pathogen susceptibility [34, 35]. No obvious phenotypic differences in growth habit, flower development or embryo morphology were detected between Fe-1 and Col-0 to suggest such a prominent difference in response to loss of ZLL function.

Although our analyses are far from saturating, most *ZLL*-dependent accessions analysed here were collected from regions within middle and southern Germany. Recent advances in SNP detection and the availability of large genomic sequence datasets from diverse accessions allows trait variation to be dissected by genome wide association studies (GWAS), which offers a much higher mapping resolution compared to the RILs [28, 36–38]. The number of accessions analysed in this experiment was insufficient for robust GWAS, but such an approach might be useful in future studies to fine map the *FHE* loci and to identify additional loci that contribute to ZLL function in stem cell maintenance. Identification of the genes underlying the *FHE* QTL will show whether the *ZLL*-dependent accessions share a common recent ancestor containing a set of genetic modifications influencing stem cell maintenance, or if geographical conditions have independently influenced selection of polymorphisms in the modifier loci.

### Conserved differences in embryonic gene expression are detected between different Arabidopsis accessions

The first defects in stem cell maintenance in *zll-1* mutants are observed at the torpedo stage of embryogenesis [10], suggesting that *FHE* modifiers of ZLL function should be active at this stage. Microarray analysis identified multiple genes showing natural variation in embryonic gene expression at the torpedo-stage. These expression profiles suggest it is unlikely that any causative polymorphisms influencing *ZLL*-dependency in the seven analysed accessions lead to common changes in mRNA expression of the genes underlying the Ler/Col *FHE* loci. This is not surprising, since the *FHE* QTL may differ between diverged accessions and the specific polymorphism(s) leading to *ZLL*-dependency may not lead to a change in mRNA expression, but rather have effects on protein function or accumulation.

Of the 12 genes differentially expressed in the combined *ZLL*-dependent versus *ZLL*-independent accessions, none were tightly linked to the Ler/Col *FHE* loci or had documented functions in meristem development or RNAi. In addition, only two of the genes showed any expression correlation across a developmental series (0.84; At1g78820 vs At5g28770; Genevestigator [39]), suggesting that the group are unlikely to be associated closely in the same pathway. Despite this, it is possible that variable expression of these genes in the different accessions is at least partly dependent on activity of the *FHE* loci. This is also possible for the remaining 427 genes that showed accession-specific expression during embryogenesis.

### A hypomorphic *SQUINT* allele may support stem cell maintenance in Columbia *zll* mutants

Restricting the embryonic expression profile comparisons to the three most relevant accessions (Col-0, Ler-0 and Fe-1), in combination with double mutant analysis, identified *SQN* as a candidate modifier underlying the *FHE2* QTL. In Col-0, embryonic *SQN* expression is 5-fold lower than Ler-0 and Fe-1. Consistent with a role in modifying stem cell development, decreased SQN activity in Ler via *sqn-4* and *amiRSQN* partially rescued stem cell maintenance in the *zll-1* background, while increased *SQN* expression in Col *zll*^*ago10-1*^ induced stem cell termination. Although the effects were greater than the predicted quantitative contribution of the *FHE2* QTL, this may be due to the nature of the polymorphisms between *SQN*^*Ler*^ and *SQN*^*Col*^*.* Only synonymous SNPs are present in the *SQN* coding sequence between Ler-0 and Col-0, indicating that differences in enzyme amino acid sequence cannot explain differences in function. In contrast, significant variations including insertions and deletions are present in the 5’ sequence upstream of the *SQN* gene ( [40]; Additional file 5). Notably, a 6.6 kb MULE-related transposon sequence, annotated as At2g15800/At2g15810, is inserted close to the transcriptional start site of *SQN*. This insertion is located 500 bp upstream of the predicted *SQN* start codon in Col-0 but is absent from Ler-0 (Additional file 5). The presence of this insertion varies between *Arabidopsis* accessions [41], and may contribute to natural variation in *SQN* expression as detected for other genes tightly linked to transposon sequences [42].

Although variable *SQN* expression levels correlate with differences in stem cell maintenance in Ler-0, Fe-1 and Col-0, and to a lesser extent in the Bay-0 and Nw-0 accessions, this is not the case in all accessions examined. *SQN* mRNA levels in the *ZLL*-independent Ts-1 and Van-0 lines were unchanged relative to Fe-1/Ler-0. Therefore, alternative *FHE* loci may play a more important role in these accessions. It is possible that some of this variation may be due to subtle transcriptional or post-transcriptional changes in the function of other meristem or RNAi-related genes physically linked to the *FHE* loci reported here (Additional file 6). Further analysis of F_2_ progeny from Ler *zll-1* and Ts-1 or Van-0 crosses will allow the major *FHE* loci that influence stem cell maintenance in these accessions to be positioned.

## Conclusions

Our current model for *FHE2* function is based upon a conserved increase in *SQN* mRNA levels in Ler-0 and Fe-1 compared to Col-0. SQN is predicted to enhance AGO1 activity through function as a co-factor [26]. In combination with a *zll* mutation, which allows AGO1 greater access to miR165/166, increased levels of *SQN* in Ler-0 enhance repression of AGO1 targets, such as the Class III HD-ZIPs, and lead to a high frequency of terminal stem cell differentiation. Conversely in Col-0, where embryonic *SQN* expression is 5-fold lower than Fe-1/Ler-0, AGO1 is less efficient at reducing Class III HD-ZIP expression and inducing stem cell termination in the absence of *ZLL* function. In line with this, Col *zll*^*ago10-1*^ mutants showed no detectable change in Class III HD-ZIP mRNA levels or other miRNA targets compared to Col-0 wild-type (Additional file 7), despite containing a functional AGO1 gene [9]. Only when embryonic *SQN* expression was increased via the *AtRPS5a:SQN* construct did a high frequency of Col *zll*^*ago10-1*^ seedlings show meristem termination. Although changes in *SQN* expression alone cannot account for the drastic differences between *zll* phenotypes in Col-0 and Ler-0, it is likely that *SQN* forms part of an important pathway that contributes to *ZLL* function and FHE activity during stem cell development. Further characterisation of the *FHE* loci using emerging genomic and genetic resources, in combination with second-site mutagenesis studies in *zll*^*ago10-1*^, will aid the identification of the responsible loci as well as determine their conservation in diverged *Arabidopsis* accessions.

## Methods

### Plant material

Seeds were germinated on soil and grown as described previously [43]. The Col *zll*^*ago10-1*^[18] and Ler *zll-1*[8] mutants have also been described previously. Seeds from the Ler/Col Recombinant Inbred Lines (N4859) and various *Arabidopsis* accessions were obtained from the Nottingham *Arabidopsis* stock centre (NASC). Single Nucleotide Polymorphism (SNP) haplotype alignments were created using publically available data (http://www.naturalvariation.org) in Geneious (http://www.geneious.com/). Maximum-likelihood trees were generated in Mega5.2 [44]. Defects in stem cell maintenance were scored in seedlings between 11–15 days post germination. Seedlings that contained an empty apex, a single filament, a single leaf or two leaves in place of a viable shoot meristem, were scored as showing stem cell defects, as per previous studies [8, 10]. Although phenotypic *zll* mutants terminate primary meristem development, secondary adventitious meristems produce viable flowers that can be used for crossing.

### Meristem measurements

The number of cells in the embryonic meristem was determined by staining with propidium iodide and confocal laser microscopy as described previously [45]. Confocal laser microscopy was performed at the Life Imaging Center (LIC, Freiburg).

### Mapping

All new markers used in this study were PCR based, and designed from the Cereon collection [46] to detect insertions/deletions (INDELs) or single nucleotide polymorphisms (SNPs) by derived cleaved amplified polymorphism (dCAPS) primers. Primer sequences are shown in Additional file 8.

### QTL analysis

Molecular map information for the Ler/Col RILs was downloaded from the NASC website (http://Arabidopsis.info/RI_data/full_markers.text). QTL analysis was performed with the software package PlabMQTL [47] using composite interval mapping [48, 49] and a multiple regression procedure [50]. Cofactors were selected based on the modified Bayesian Information Criterion [51] and critical Logarithm of the odds (LOD) thresholds were determined empirically with 1,000 random permutations [52]. The proportion of variance explained by the detected QTL (*p*) was obtained from the adjusted R^2^ value of the QTL model and the proportion of variance explained by individual QTL by normalizing to sum up to the total *p*.

### Microarray profiling

Torpedo-stage embryos were dissected from maturing seeds in 1×PBS and stored on ice for no longer than 1 hour before snap freezing in liquid nitrogen. Approximately 100 embryos were harvested from each accession. RNA was extracted using the RNeasy Plant Minikit (with on-column DNAse treatment; Qiagen) according to manufacturer’s instructions. 10 μg total RNA was hybridised to Affymetrix ATH1 chips at ATLAS Biolabs (Berlin, Germany). Expression analysis and normalisation was performed in R using the RMA package, following a previously established pipeline [53]

### Cloning

Three artificial miRNAs targeting *SQN* were designed using the Web MicroRNA designer program; http://wmd3.weigelworld.org/cgi-bin/webapp.cgi[54] and cloned into the pJet2.1 expression vector. After sequencing, the amiRNA constructs were sub-cloned via BamHI digest into pEG278. Finally, *p35S:amiRSQN-term* was cloned into the PacI site of the pGreen II destination vector [55]. Plant transformation was carried out by Agrobacterium-mediated floral dipping. After selection of T1 and T2 transformants, T3 homomozygous lines were used for final analysis.

To generate *pAtRPS5a:SQN,* genomic DNA of *SQN* was amplified by oFR126-Fr/oFR127-Rev primers harboring LIC cloning fragments and subcloned into the pJet2.1 expression vector. After sequencing, the *SQN* genomic fragment was cloned using the LIC cloning protocol [56], into a modified pGreenII vector containing the *AtRPS5a* promoter, LIC cloning site and *Nos* terminator. Full primer details are available on request.

### Availability of supporting data

The microarray data sets supporting the results of this article are available in the Gene Expression Omnibus (GEO) repository, accessible via the GSE47884 identifier.

## Electronic supplementary material

Additional file 1: **Table showing the frequency of shoot meristem termination phenotypes in** ***zll-1*** **x Ler/Col RIL F**_**2**_ **seedlings as a proportion of the total homozygous mutants.** (PDF 50 KB)

Additional file 2: **Chromosome map showing INDEL and dCAPs markers used for near isogenic line genotyping.** (PDF 633 KB)

Additional file 3: **Table showing total number of genes differentially expressed (log**_**2**_ **FC >1.5, p <0.05) in torpedo stage embryos from different** ***Arabidopsis*** **accessions.** (PDF 55 KB)

Additional file 4: **Table showing differentially expressed genes between all** ***ZLL*** **-dependent and** ***ZLL*** **-independent accessions.** (PDF 58 KB)

Additional file 5: **Table showing polymorphisms identified between the Ler-0 and Col-0 genomic sequence of** ***SQN*** [40]. (XLSX 23 KB)

Additional file 6: **Table showing meristem and RNAi-related genes located close to** ***FHE*** **QTL map positions.** (PDF 64 KB)

Additional file 7: **Table showing genes with altered expression in** ***zll*** ^***ago10-1***^ **inflorescence meristems compared to Col-0.** (PDF 85 KB)

Additional file 8: **Table showing primer sequences used for mapping.** (PDF 51 KB)

## References

[1] Aichinger E, Kornet N, Friedrich T, Laux T (2012). Plant stem cell niches. Annu Rev Plant Biol.

[2] Murray JA, Jones A, Godin C, Traas J (2012). Systems analysis of shoot apical meristem growth and development: integrating hormonal and mechanical signaling. Plant Cell.

[3] Tucker MR, Laux T (2007). Connecting the paths in plant stem cell regulation. Trends Cell Biol.

[4] Knauer S, Holt AL, Rubio-Somoza I, Tucker EJ, Hinze A, Pisch M, Javelle M, Timmermans MC, Tucker MR, Laux T (2013). A protodermal miR394 signal defines a region of stem cell competence in the arabidopsis shoot meristem. Dev Cell.

[5] Mayer KFX, Schoof H, Haecker A, Lenhard M, Jürgens G, Laux T (1998). Role of WUSCHEL in regulating stem cell fate in the Arabidopsis shoot meristem. Cell.

[6] Schoof H, Lenhard M, Haecker A, Mayer KFX, Jürgens G, Laux T (2000). The stem cell population of Arabidopsis shoot meristems is maintained by a regulatory loop between the CLAVATA and WUSCHEL genes. Cell.

[7] Brand U, Fletcher JC, Hobe M, Meyerowitz EM, Simon R (2000). Dependence of stem cell fate in Arabidopsis on a feedback loop regulated by CLV3 activity. Science.

[8] Moussian B, Schoof H, Haecker A, Jürgens G, Laux T (1998). Role of the ZWILLE gene in the regulation of central shoot meristem cell fate during Arabidopsis embryogenesis. EMBO J.

[9] Bohmert K, Camus I, Bellini C, Bouchez D, Caboche M, Benning C (1998). AGO1 defines a novel locus of Arabidopsis controlling leaf development. EMBO J.

[10] Tucker MR, Hinze A, Tucker EJ, Takada S, Jurgens G, Laux T (2008). Vascular signalling mediated by ZWILLE potentiates WUSCHEL function during shoot meristem stem cell development in the Arabidopsis embryo. Development.

[11] Mallory AC, Hinze A, Tucker MR, Bouche N, Gasciolli V, Elmayan T, Lauressergues D, Jauvion V, Vaucheret H, Laux T (2009). Redundant and specific roles of the ARGONAUTE proteins AGO1 and ZLL in development and small RNA-directed gene silencing. PLoS Genet.

[12] Zhu H, Hu F, Wang R, Zhou X, Sze SH, Liou LW, Barefoot A, Dickman M, Zhang X (2011). Arabidopsis Argonaute10 specifically sequesters miR166/165 to regulate shoot apical meristem development. Cell.

[13] Lynn K, Fernandez A, Aida M, Sedbrook J, Tasaka M, Masson P, Barton MK (1999). The PINHEAD/ZWILLE gene acts pleiotropically in Arabidopsis development and has overlapping functions with the ARGONAUTE1 gene. Development.

[14] Brodersen P, Sakvarelidze-Achard L, Bruun-Rasmussen M, Dunoyer P, Yamamoto YY, Sieburth L, Voinnet O (2008). Widespread translational inhibition by plant miRNAs and siRNAs. Science.

[15] Vaucheret H (2008). Plant ARGONAUTES. Trends Plant Sci.

[16] Manavella PA, Weigel D, Wu L (2011). Argonaute10 as a miRNA locker. Cell.

[17] Liu Q, Yao X, Pi L, Wang H, Cui X, Huang H (2008). The ARGONAUTE10 gene modulates shoot apical meristem maintenance and leaf polarity establishment by repressing miR165/166 in Arabidopsis. Plant J.

[18] Takeda A, Iwasaki S, Watanabe T, Utsumi M, Watanabe Y (2008). The mechanism selecting the guide strand from small RNA duplexes is different among argonaute proteins. Plant Cell Physiol.

[19] Vollbrecht E, Reiser L, Hake S (2000). Shoot meristem size is dependent on inbred background and presence of the maize homeobox gene, knotted1. Development.

[20] Moussian B, Haecker A, Laux T (2003). ZWILLE buffers meristem stability in Arabidopsis thaliana. Dev Genes Evol.

[21] Torii KU, Mitsukawa N, Oosumi T, Matsuura Y, Yokoyama R, Whittier RF, Komeda Y (1996). The Arabidopsis ERECTA gene encodes a putative receptor protein kinase with extracellular leucine-rich repeats. Plant Cell.

[22] Platt A, Horton M, Huang YS, Li Y, Anastasio AE, Mulyati NW, Agren J, Bossdorf O, Byers D, Donohue K (2010). The scale of population structure in Arabidopsis thaliana. PLoS Genet.

[23] Lister C, Dean C (1993). Recombinant inbred lines for mapping rflp and phenotypic markers in arabidopsis-thaliana. Plant J.

[24] Berardini TZ, Bollman K, Sun H, Poethig RS (2001). Regulation of vegetative phase change in Arabidopsis thaliana by cyclophilin 40. Science.

[25] Prunet N, Morel P, Thierry AM, Eshed Y, Bowman JL, Negrutiu I, Trehin C (2008). REBELOTE, SQUINT, and ULTRAPETALA1 function redundantly in the temporal regulation of floral meristem termination in Arabidopsis thaliana. Plant Cell.

[26] Smith MR, Willmann MR, Wu G, Berardini TZ, Moller B, Weijers D, Poethig RS (2009). Cyclophilin 40 is required for microRNA activity in Arabidopsis. Proc Natl Acad Sci USA.

[27] Weigel D (2012). Natural variation in Arabidopsis: from molecular genetics to ecological genomics. Plant Physiol.

[28] Atwell S, Huang YS, Vilhjalmsson BJ, Willems G, Horton M, Li Y, Meng D, Platt A, Tarone AM, Hu TT (2010). Genome-wide association study of 107 phenotypes in Arabidopsis thaliana inbred lines. Nature.

[29] Zhai J, Liu J, Liu B, Li P, Meyers BC, Chen X, Cao X (2008). Small RNA-directed epigenetic natural variation in Arabidopsis thaliana. PLoS Genet.

[30] Nasrallah ME, Liu P, Sherman-Broyles S, Boggs NA, Nasrallah JB (2004). Natural variation in expression of self-incompatibility in Arabidopsis thaliana: implications for the evolution of selfing. Proc Natl Acad Sci USA.

[31] Mouchel CF, Briggs GC, Hardtke CS (2004). Natural genetic variation in Arabidopsis identifies BREVIS RADIX, a novel regulator of cell proliferation and elongation in the root. Genes Dev.

[32] Lall S, Nettleton D, DeCook R, Che P, Howell SH (2004). Quantitative trait loci associated with adventitious shoot formation in tissue culture and the program of shoot development in Arabidopsis. Genet.

[33] DeCook R, Lall S, Nettleton D, Howell SH (2006). Genetic regulation of gene expression during shoot development in Arabidopsis. Genet.

[34] Wang Y, Meng Y, Zhang M, Tong X, Wang Q, Sun Y, Quan J, Govers F, Shan W (2011). Infection of Arabidopsis thaliana by Phytophthora parasitica and identification of variation in host specificity. Mol Plant Pathol.

[35] Grant MR, McDowell JM, Sharpe AG, de Torres ZM, Lydiate DJ, Dangl JL (1998). Independent deletions of a pathogen-resistance gene in Brassica and Arabidopsis. Proc Natl Acad Sci USA.

[36] Seren U, Vilhjalmsson BJ, Horton MW, Meng D, Forai P, Huang YS, Long Q, Segura V, Nordborg M (2012). GWAPP: a Web application for genome-wide association mapping in arabidopsis. Plant Cell.

[37] Cockram J, White J, Zuluaga DL, Smith D, Comadran J, Macaulay M, Luo Z, Kearsey MJ, Werner P, Harrap D (2010). Genome-wide association mapping to candidate polymorphism resolution in the unsequenced barley genome. Proc Natl Acad Sci USA.

[38] Alheit KV, Maurer HP, Reif JC, Tucker MR, Hahn V, Weissmann EA, Wurschum T (2012). Genome-wide evaluation of genetic diversity and linkage disequilibrium in winter and spring triticale (x Triticosecale Wittmack). BMC Genomics.

[39] Hruz T, Laule O, Szabo G, Wessendorp F, Bleuler S, Oertle L, Widmayer P, Gruissem W, Zimmermann P (2008). Genevestigator v3: a reference expression database for the meta-analysis of transcriptomes. Adv Bioinf.

[40] Gan X, Stegle O, Behr J, Steffen JG, Drewe P, Hildebrand KL, Lyngsoe R, Schultheiss SJ, Osborne EJ, Sreedharan VT (2011). Multiple reference genomes and transcriptomes for Arabidopsis thaliana. Nature.

[41] Cao J, Schneeberger K, Ossowski S, Gunther T, Bender S, Fitz J, Koenig D, Lanz C, Stegle O, Lippert C (2011). Whole-genome sequencing of multiple Arabidopsis thaliana populations. Nat Genet.

[42] Wang X, Weigel D, Smith LM (2013). Transposon variants and their effects on gene expression in Arabidopsis. PLoS Genet.

[43] Laux T, Mayer KFX, Berger J, Jürgens G (1996). The WUSCHEL gene is required for shoot and floral meristem integrity in Arabidopsis. Development.

[44] Tamura K, Peterson D, Peterson N, Stecher G, Nei M, Kumar S (2011). MEGA5: molecular evolutionary genetics analysis using maximum likelihood, evolutionary distance, and maximum parsimony methods. Mol Biol Evol.

[45] Wurschum T, Gross-Hardt R, Laux T (2006). APETALA2 regulates the stem cell niche in the Arabidopsis shoot meristem. Plant Cell.

[46] Jander G, Norris SR, Rounsley SD, Bush DF, Levin IM, Last RL (2002). Arabidopsis map-based cloning in the post-genome era. Plant Physiol.

[47] Utz HF (2012). PlabMQTL Manual - Software for meta-QTL analysis with composite interval mapping. Version 0.5s.

[48] Jansen RC, Stam P (1994). High resolution of quantitative traits into multiple loci via interval mapping. Genet.

[49] Zeng ZB (1994). Precision mapping of quantitative trait loci. Genet.

[50] Haley CS, Knott SA (1992). A simple regression method for mapping quantitative trait loci in line crosses using flanking markers. Heredity (Edinb).

[51] Baierl A, Bogdan M, Frommlet F, Futschik A (2006). On locating multiple interacting quantitative trait loci in intercross designs. Genet.

[52] Churchill GA, Doerge RW (1994). Empirical threshold values for quantitative trait mapping. Genet.

[53] D’Onofrio C, Cox A, Davies C, Boss PK (2009). Induction of secondary metabolism in grape cell cultures by jasmonates. Funct Plant Biol.

[54] Schwab R, Ossowski S, Riester M, Warthmann N, Weigel D (2006). Highly specific gene silencing by artificial microRNAs in Arabidopsis. Plant Cell.

[55] Hellens R, Mullineaux P, Klee H (2000). Technical focus:a guide to agrobacterium binary Ti vectors. Trends Plant Sci.

[56] Eschenfeldt WH, Lucy S, Millard CS, Joachimiak A, Mark ID (2009). A family of LIC vectors for high-throughput cloning and purification of proteins. Methods Mol Biol.

