# Supplementary material for: Accession-specific modifiers act with ZWILLE/ARGONAUTE10 to maintain shoot meristem stem cells during embryogenesis in Arabidopsis
Source: BMC Genomics. 2013 Nov 20;14(1):809. doi: 10.1186/1471-2164-14-809 (PMC4046819; doi:10.1186/1471-2164-14-809)
Supplement: Supplementary file 1 — Additional file 1: Table showing the frequency of shoot meristem termination phenotypes in zll-1 x Ler/Col RIL F 2 seedlings as a proportion of the total homozygous mutants. (PDF 50 KB) [file 12864_2013_5527_MOESM1_ESM.pdf]

Additional File 1: Shoot meristem termination in *zll-1* x Ler/Col RIL F_2 seedlings as a proportion of the total homozygous mutants.

RIL Identifier	% Seedlings showing meristem termination	RIL Identifier	% Seedlings showing meristem termination	RIL Identifier	% Seedlings showing meristem termination
Col	2	N1933	-	N1967	-
Ler	55	N1934	16	N1968	33
N1900	8	N1935	29	N1969	33
N1901	6	N1936	17	N1970	0
N1903	5	N1937	-	N1971	13
N1904	21	N1938	40	N1972	47
N1905	15	N1939	25	N1973	11
N1906	9	N1940	7	N1974	37
N1907	43	N1941	11	N1975	7
N1908	27	N1942	43	N1976	20
N1909	9	N1943	60	N1977	34
N1910	28	N1944	14	N1978	28
N1911	30	N1945	22	N1979	8
N1912	25	N1946	14	N1980	42
N1913	17	N1947	6	N1981	11
N1914	13	N1948	19	N1982	7
N1915	7	N1949	12	N1983	14
N1916	10	N1950	21	N1984	17
N1917	32	N1951	7	N1985	6
N1918	21	N1952	-	N1986	27
N1919	9	N1953	28	N1987	18
N1920	13	N1954	20	N1988	16
N1921	14	N1955	5	N1989	19
N1922	27	N1956	13	N1990	34
N1923	59	N1957	21	N1991	16
N1924	13	N1958	27	N1992	24
N1925	16	N1959	29	N1993	4
N1926	27	N1960	22	N1994	30
N1927	16	N1961	7	N1995	6
N1928	23	N1962	36	N1996	20
N1929	10	N1963	44	N1997	13
N1930	18	N1964	30	N1998	11
N1931	42	N1965	19	N1999	10
N1932	19	N1966	18	N4686	38
