# Supplementary material for: Accession-specific modifiers act with ZWILLE/ARGONAUTE10 to maintain shoot meristem stem cells during embryogenesis in Arabidopsis
Source: BMC Genomics. 2013 Nov 20;14(1):809. doi: 10.1186/1471-2164-14-809 (PMC4046819; doi:10.1186/1471-2164-14-809)
Supplement: Supplementary file 2 — Additional file 2: Chromosome map showing INDEL and dCAPs markers used for near isogenic line genotyping. (PDF 633 KB) [file 12864_2013_5527_MOESM2_ESM.pdf]

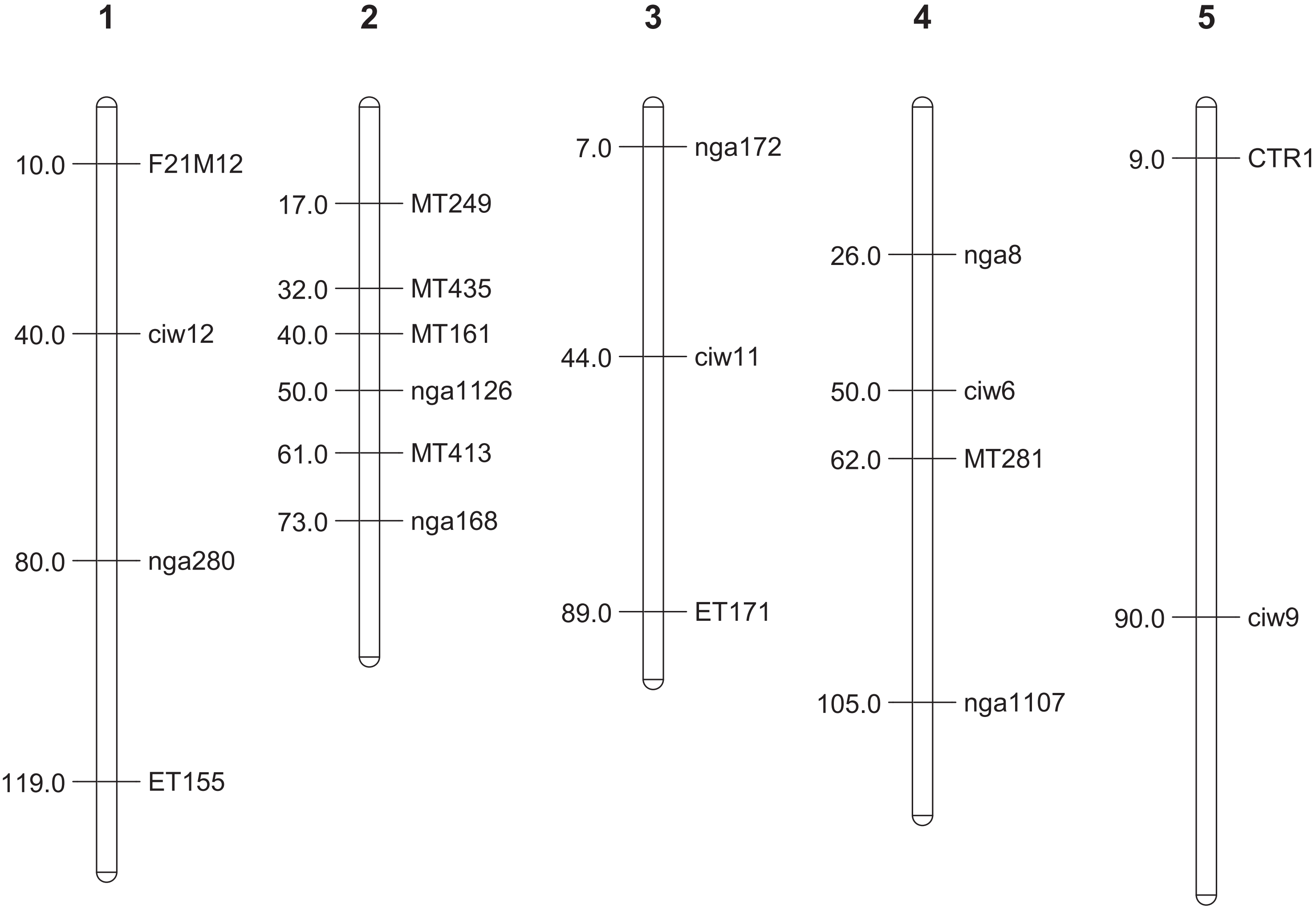

Additional File 2. Insertion/Deletion (INDEL) and derived Cleaved Amplified Polymorphic Sequence (dCAPs) markers used for near isogenic line genotyping. Numbers at the top indicate the chromosome while numbers on chromosomes indicate the centiMorgan (cM) position.
