# Supplementary material for: Accession-specific modifiers act with ZWILLE/ARGONAUTE10 to maintain shoot meristem stem cells during embryogenesis in Arabidopsis
Source: BMC Genomics. 2013 Nov 20;14(1):809. doi: 10.1186/1471-2164-14-809 (PMC4046819; doi:10.1186/1471-2164-14-809)
Supplement: Supplementary file 4 — Additional file 4: Table showing differentially expressed genes between all ZLL -dependent and ZLL -independent accessions. (PDF 58 KB) [file 12864_2013_5527_MOESM4_ESM.pdf]

Additional File 4. Genes showing differential expression between grouped *ZLL*-dependent and *ZLL*-independent accessions.

**Affy ID**	**Gene**	**Description**	**Fold Change (ZLL-dep. vs ZLL-ind.)**	**p-value**
246471_s_at	AT5G17100	unknown	-10.0	0.00
246817_at	AT5G27240	DNAJ domain-containing protein	-3.3	0.01
247988_at	AT5G56910	unknown	3.0	0.01
245925_at	AT5G28770	BZO2H3; DNA binding	3.2	0.01
245442_at	AT4G16710	Glycosyl transferase 28	3.2	0.02
267110_at	AT2G14800	unknown	3.7	0.01
264279_s_at	AT1G78820	curculin-like lectin family protein	4.6	0.02
251347_at	AT3G61010	Glycosyl hydrolase 85	4.6	0.04
245169_at	AT2G33220	Similar to MEE4	5.3	0.00
250038_at	AT5G18360	Putative TIR-NBS-LRR	5.7	0.00
255939_at	AT1G12730	cell division cycle protein-related	6.1	0.00
249479_at	AT5G38960	Putative germin-like protein	6.5	0.00

Affy ID = Affymetrix gene chip identifier, ZLL-dep. = ZLL-dependent, ZLL-ind. = ZLL independent, Fold change = >3.0, p <0.05
