# Supplementary material for: Accession-specific modifiers act with ZWILLE/ARGONAUTE10 to maintain shoot meristem stem cells during embryogenesis in Arabidopsis
Source: BMC Genomics. 2013 Nov 20;14(1):809. doi: 10.1186/1471-2164-14-809 (PMC4046819; doi:10.1186/1471-2164-14-809)
Supplement: Supplementary file 6 — Additional file 6: Table showing meristem and RNAi-related genes located close to FHE QTL map positions. (PDF 64 KB) [file 12864_2013_5527_MOESM6_ESM.pdf]

Additional File 6. Meristem and RNAi-related genes located close to *FHE* QTL map positions.

QTL	Gene	Description
FHE1	AT1G46480	WOX4 (WUSCHEL-RELATED HOMEOBOX 4)
	AT1G48267	miR161
	AT1G48410	AGO1 (ARGONAUTE 1)
	AT1G48742	miR157D
	AT1G49190	ARR19 (*ARABIDOPSIS* RESPONSE REGULATOR 19)
	AT1G50030	TOR (TARGET OF RAPAMYCIN)
FHE2	AT2G15790	SQN (SQUINT)
	AT2G16145	miR416
	AT2G16390	DRD1 (DEFECTIVE IN RNA-DIRECTED DNA METHYLATION 1)
FHE3	AT2G28610	PRS (PRESSED FLOWER); transcription factor
	AT2G28650	ATEXO70H8 (exocyst subunit EXO70 family protein H8)
	AT2G32940	ARGONAUTE6 (AGO6)
	AT2G33480	ANAC041 (*Arabidopsis* NAC domain containing protein 41)
	AT2G33860	ETT (ETTIN); transcription factor
	AT2G33880	WOX9 (STIMPY); transcription factor
	AT2G34202	miR399D
	AT2G34204	miR399E
	AT2G34208	miR399F
	AT2G34650	PID (PINOID); kinase
	AT2G34710	PHB (PHABULOSA); DNA binding / transcription factor
	AT2G35350	PLL1 (POLTERGESIST-LIKE 1)
	AT2G36490	DML1/ROS1 (REPRESSOR OF SILENCING1)
	AT2G36890	ATMYB38/MYB38/RAX2 (myb domain protein 38)
FHE4	AT4G35550	HB-4/WOX13 (WUSCHEL-RELATED HOMEOBOX 13)
	AT4G36920	AP2 (APETALA 2)
	AT4G37510	ribonuclease III family protein
FHE5	-	-
