# Supplementary material for: Accession-specific modifiers act with ZWILLE/ARGONAUTE10 to maintain shoot meristem stem cells during embryogenesis in Arabidopsis
Source: BMC Genomics. 2013 Nov 20;14(1):809. doi: 10.1186/1471-2164-14-809 (PMC4046819; doi:10.1186/1471-2164-14-809)
Supplement: Supplementary file 7 — Additional file 7: Table showing genes with altered expression in zll ago10-1 inflorescence meristems compared to Col-0. (PDF 85 KB) [file 12864_2013_5527_MOESM7_ESM.pdf]

Additional File 7. Genes with altered expression in *zll*$^{ago10\text{-}1}$ inflorescence meristems compared to Columbia-0.

Gene	Fold Change	p-value	Name	Gene Function	Col-0 (3 replicate average)	SD	*ago10-1* (3 replicate average)	SD
AT5G43810	-12	0	ZLL	ZLL (ZWILLE) ARGONAUTE Protein	11194	622	1017	476
AT3G30720	-5.5	0.017		unknown protein	3048	844	540	95
AT1G17150	-3.8	0.04		polygalacturonase (pectinase) family protein	17	3	7	6
AT3G45670	-3	0.002		protein kinase-related	235	24	79	7
AT3G01530	-2.9	0.015	AtMYB57	DNA binding / transcription factor	1929	493	674	234
AT2G33830	-2.9	0.018		dormancy/auxin associated family protein	2920	668	980	86
AT4G12490	-2.9	0.04		lipid transfer protein (LTP) family protein	24	9	9	5
AT4G12530	-2.8	0.003		lipid transfer protein (LTP) family protein	729	73	256	20
AT1G66100	-2.8	0.046		thionin, putative	53	20	18	5
AT5G28897	-2.8	0.021		pseudogene	22	6	7	2
AT2G47270	-2.8	0.049		transcription factor/ transcription regulator	233	95	83	31
AT1G58037	-2.8	0.037		similar to DC1 domain-containing protein	22	3	10	6
AT3G48340	-2.8	0.004		cysteine-type peptidase	255	22	97	39
AT1G29660	-2.6	0		GDSL-motif lipase/hydrolase family protein	1272	64	487	81
AT3G04720	-2.6	0.031	PR4	PATHOGENESIS-RELATED 4	663	192	248	27
AT3G27940	-2.6	0.017	LBD26	LOB DOMAIN-CONTAINING 26	153	31	59	3
AT2G24210	-2.5	0.049	TPS10	TERPENE SYNTHASE 10	8784	3351	3421	1117
AT2G36790	-2.5	0.019	UGT73C6	UDP-glucosyltransferase	137	36	56	19
AT5G55690	-2.5	0.045	AGL47	MADS-box protein	415	141	163	7
AT1G29830	-2.5	0.013		unknown	108	21	44	6
AT2G37720	-2.4	0.029		unknown	24	7	10	3
AT1G62280	-2.4	0.021	SLAH1	transporter	126	27	57	30
AT5G38970	-2.4	0.045	BR6OX1	BRASSINOSTEROID-6-OXIDASE	906	301	413	221
AT4G35770	-2.3	0.041	SEN1	DARK INDUCIBLE 1	529	173	219	42
AT2G17000	-2.3	0.038		MS ion channel domain-containing protein	87	28	37	10
AT5G44020	-2.3	0.037		acid phosphatase class B family protein	13150	3957	5584	887
AT3G32980	-2.3	0.022	PER32	peroxidase 32 (PER32)	21	1	10	5
AT2G40113	-2.3	0.009		unknown	39	2	18	6
AT1G78850	-2.3	0.038		lectin family protein	124	39	53	12
AT3G10320	-2.3	0.025	DUF563	unknown	1514	382	694	328
AT3G15450	-2.3	0.035		unknown	65486	18623	28245	2906
AT3G14220	-2.2	0.001		GDSL-motif lipase/hydrolase family protein	2829	259	1274	227
AT1G49500	-2.2	0.01		unknown	443	80	200	38
AT1G47790	-2.2	0.045		F-box family protein	16	5	8	5
AT5G04200	-2.2	0.018	ATMC9	caspase/ cysteine-type peptidase	936	111	453	204
AT1G20180	-2.2	0.045		unknown	232	77	103	22
AT3G01420	-2.2	0.017		ALPHA-DIOXYGENASE 1	95	19	43	5
AT1G61560	-2.2	0.024	MLO6	MILDEW RESISTANCE LOCUS O 6	446	103	217	98
AT5G20250	-2.2	0.041	DIN10	DARK INDUCIBLE 10; hydrolase	10689	3260	4815	778
AT2G01280	-2.2	0.021	MEE65	RNA polymerase II	73	16	34	5
AT1G27020	-2.2	0.002		unknown	265	29	125	27
AT4G28040	-2.1	0.011		nodulin MtN21 family protein	536	102	254	68
AT1G09155	-2.1	0.036	ATPP2-B15	Phloem protein 2-B15; carbohydrate binding	291	86	139	56
AT2G43820	-2.1	0.025	UGT74F2	UDP-glucosyltransferase	829	193	386	52
AT4G35060	-2.1	0.041		heavy-metal-associated protein	1678	481	838	398

AT3G20940	-2.1	0.027	CYP705A30	cytochrome P450	415	57	212	103
AT4G15760	-2.1	0.013		monooxygenase	1339	247	641	95
AT3G59850	-2.1	0.013		polygalacturonase	201	40	97	23
AT3G47210	-2.1	0.004		unknown	118	13	56	4
AT5G25205	-2.1	0.008		transposable element gene	14	3	7	2
AT3G22250	-2.1	0.028		UDP-glucoronosyl/UDP-glucosyl transferase	124	31	62	24
AT3G08040	-2.1	0.009	FRD3	(FERRIC REDUCTASE DEFECTIVE 3	808	63	407	129
AT1G53830	-2.1	0.034	ATPME2	*Arabidopsis* thaliana pectin methylesterase 2	357	49	187	93
AT1G10455	-2.1	0.023		unknown	30	6	15	7
AT2G38380	-2	0.043		peroxidase 22 (PER22)	80	23	38	5
AT2G15345	-2	0.025		enzyme inhibitor/ pectinesterase	19	4	10	4
AT1G63030	-2	0.021	DDF2	DWARF AND DELAYED FLOWERING	35	5	18	7
AT5G56970	-2	0.011	CKX3	CYTOKININ OXIDASE 3	2286	417	1138	266
AT5G27845	-2	0.029		transposable element gene	21	5	10	1
AT4G39780	-2	0.033		AP2 domain-containing transcription factor	2119	520	1034	69
AT4G37800	-2	0.027		xyloglucan:xyloglucosyl transferase	3483	846	1724	333
AT2G18200	-2	0.044		unknown	27	8	14	6
AT1G22400	-2	0.048	UGT85A1	UDP-glycosyltransferase	4326	1174	2298	1112
AT4G15590	-2	0.049		transposable element gene	19	6	10	4
AT5G35354	-2	0.014		transposable element gene	11	2	6	2
AT4G37610	-2	0.014	BT5	BTB and TAZ domain protein 5	3953	742	2000	349
AT5G34858	-2	0.014		transposable element gene	34	6	17	1
AT1G70680	-2	0.016		caleosin-related family protein	267	50	140	46
AT5G56920	2	0.014		cysteine protease inhibitor	8	2	15	3
AT3G60610	2	0.034		pseudogene	13	6	24	4
AT4G30380	2	0.007	EXLB2	EXPANSIN-LIKE B2 PRECURSOR	14	3	26	4
AT2G03370	2	0.04		serine carboxypeptidase	26	12	48	11
AT2G11851	2	0.033		unknown	9	4	17	1
AT3G01700	2	0.018	AGP11	ARABINOGALACTAN PROTEIN 11	2072	766	3973	736
AT1G18140	2	0	LAC1	Laccase 1; copper ion binding	2562	222	5107	337
AT4G36430	2	0.001		peroxidase	101	17	201	10
AT1G15640	2	0.02		unknown	17	7	32	4
AT4G15210	2	0.002		BETA-AMYLASE	21826	4720	44043	3072
AT1G71450	2	0.045		AP2 domain-containing transcription factor	16	6	33	10
AT1G66020	2	0.016		terpene synthase/cyclase family protein	13	3	26	6
AT5G30520	2.1	0.008		unknown	7	3	14	1
AT5G03400	2.1	0.031		unknown	16	8	30	6
AT3G08900	2.1	0.008	RGP3	Reversibly Glycosylated Polypeptide 3	611	193	1232	177
AT1G29860	2.1	0.036	WRKY71	WRKY DNA-binding protein 71	7	3	13	2
AT5G32566	2.1	0.019		transposable element gene	194	81	383	41
AT5G22610	2.1	0.028		F-box family protein	45	21	86	13
AT3G31430	2.1	0.019		unknown	10	4	20	2
AT4G31877	2.1	0.009	MIR156C	miRNA	24	2	51	8
AT4G08560	2.1	0.047	APUM15	*ARABIDOPSIS* PUMILIO 15 RNA binding	6	3	11	2
AT5G56470	2.1	0.026		FAD-dependent oxidoreductase	18	9	36	3
AT1G23110	2.1	0.047		unknown	149	14	330	105
AT3G20590	2.1	0.026		non-race specific disease resistance protein	683	341	1372	203
AT1G59885	2.2	0.046		unknown	8	5	16	4
AT1G50580	2.2	0.001		glycosyltransferase family protein	55	7	120	11
AT3G42130	2.2	0.039		glycine-rich protein	1392	771	2748	307
AT2G25160	2.2	0.018	CYP82F1	cytochrome P450, family 82	13	6	27	6
AT3G05920	2.3	0.05		heavy-metal-associated protein	361	216	710	178
AT1G26870	2.3	0.03	ANAC009	*Arabidopsis* NAC domain containing protein	16	8	32	3
AT4G11190	2.3	0.003		disease resistance-responsive family protein	522	159	1186	152

AT2G30210	2.3	0.033	LAC3	laccase 3; copper ion binding	24	14	49	9
AT5G28600	2.4	0.036		transposable element gene	15	7	35	11
AT1G63950	2.4	0.019		heavy-metal-associated protein	26	4	63	14
AT4G19920	2.7	0.009		disease resistance protein (TIR class)	5	2	12	2
AT3G19350	2.7	0.025		polyadenylate-binding protein-related	72	24	189	55
AT5G28615	2.8	0.048		unknown	30	24	70	19
AT5G35610	3.2	0.004		paired amphipathic helix repeat-containing	12	5	36	7
AT2G11405	4.7	0.002		unknown	15	1	68	6

Legend: SD = standard deviation,
