# Supplementary material for: Accession-specific modifiers act with ZWILLE/ARGONAUTE10 to maintain shoot meristem stem cells during embryogenesis in Arabidopsis
Source: BMC Genomics. 2013 Nov 20;14(1):809. doi: 10.1186/1471-2164-14-809 (PMC4046819; doi:10.1186/1471-2164-14-809)
Supplement: Supplementary file 8 — Additional file 8: Table showing primer sequences used for mapping. (PDF 51 KB) [file 12864_2013_5527_MOESM8_ESM.pdf]

Additional File 8: PCR markers used for mapping QTL.

Marker Name	ID	Chr	Pos (cM)	Forward Primer	Reverse Primer	INDEL (bp Col/Ler)
F21M12	-	1	11	GGCTTTCTCGAAATCTGTCC	TTACTTTTTGCCTCTTGTCATTG	160/200
ciw12	-	1	40	AGGTTTTATTGCTTTTCACA	CTTTCAAAAGCACATCACA	128/115
nga280	-	1	83	GGCTCCATAAAAAGTGCACC	CTGATCTCACGGACAATAGTGC	105/85
ET155	CER450872	1	119	AAGTCCCGAGAATTTACCCA	GGGTTTTCAGAGAGTGATGATG	215/232
MT249	-	2	17	CAAAAGGCGAAACTCGACTGTA	CGTAGCCTTGTGGCTTCTCGGT	160/130
MT435	CER452622	2	32	TCTTCGGAGGATTACTTTGATGC	TGATGATGACAACAGGGTCGTT	150/135
MT161	CER459796	2	40	AGGTGAAGATCTCGCGCTAAT	ACTGTTTTAATCGAAGATGGATGTTA	150/162
nga1126	-	2	50	CGCTACGCTTTTCGGTAAAG	GCACAGTCCAAGTCACAACC	199/191
MT413	CER458055	2	61	TCTTTCCCATTGTGCACTATCTTG	CGACACCTTTTTAATCGCAAGTAAG	150/135
nga168	-	2	73	TCGTCTACTGCACTGCCG	GAGGACATGTATAGGAGCCTCG	135/151
nga172	-	3	7	CATCCGAATGCCATTGTTC	AGCTGCTTCCTTATAGCGTCC	162/136
ciw11	-	3	43	CCCCGAGTTGAGGTATT	GAAGAAATTCCTAAAGCATTC	230/179
ET171	CER479303	3	89	ATTGAAGTTGCACGATCATGCG	TCCAAACCATGCAATGATGC	251/226
nga8	-	4	26	TGGCTTTCGTTTATAAACATCC	GAGGGCAAATCTTTATTTCGG	154/198
ciw6	-	4	47	CTCGTAGTGCACTTTCATCA	CACATGGTTAGGGAAACAATA	148/162
MT281	-	4	62	TGTTTCAGAGTAGCCAATTC	CATCCATCAAACAAACTCC	200/160
nga1107	-	4	104	GCGAAAAAACAAAAAAATCCA	CGACGAATCGACAGAATTAGG	140/150
CTR1	-	5	10	CCACTTGTTTCTCTCTCTAG	TATCAACAGAAACGCACCGAG	143/159
ciw9	-	5	88	CAGACGTATCAAATGACAAATG	GACTACTGCTCAAACTATTCGG	145/165

ID = Cereon database marker, Pos (cM) = approximate centiMorgan position, INDEL = insertion/deletion
