# Supplementary material for: Accession-specific modifiers act with ZWILLE/ARGONAUTE10 to maintain shoot meristem stem cells during embryogenesis in Arabidopsis
Source: BMC Genomics. 2013 Nov 20;14(1):809. doi: 10.1186/1471-2164-14-809 (PMC4046819; doi:10.1186/1471-2164-14-809)
Supplement: Supplementary file 3 — Additional file 3: Table showing total number of genes differentially expressed (log 2 FC >1.5, p <0.05) in torpedo stage embryos from different Arabidopsis accessions. (PDF 55 KB) [file 12864_2013_5527_MOESM3_ESM.pdf]

Additional File 2. Total number of genes differentially expressed (FC >3.0, p <0.05) in torpedo stage embryos from different *Arabidopsis* accessions.

	**Fe-1**	**Fe-1/ Ler-1**	**Fe-1/Nw-1/Bay-0**	**Fe-1/Ler-0/Nw-0**	**Fe-1/Ler-1/ Bay-0**	**Ler-0/Nw-0/ Bay-0**	**Fe-1/Ler-0/Nw-0/Bay-0**
**Col-0**	385	184	189	126	155	80	109
**Col-0/Ts-1**	236	99	89	51	66	18	53
**Col-0/Van-0**	209	60	69	18	27	5	18
**Col-0/Ts-1 /Van-0**	172	31	52	17	24	7	12

Fe = Freiburg-1, Ler = Landsberg *erecta*-0, Nw =Neuweilnau-0, Bay = Bayreuth-0, Col = Columbia-0, Ts = Tossa del Mar-1, Van = Vancouver-0.
